# Management of Posterior Fossa Tumors in Adults Based on the Predictors of Postoperative Hydrocephalus

**DOI:** 10.3389/fsurg.2022.886438

**Published:** 2022-06-01

**Authors:** Chengda Zhang, Tingbao Zhang, Lingli Ge, Zhengwei Li, Jincao Chen

**Affiliations:** ^1^ Department of Neurosurgery, Zhongnan Hospital of Wuhan University, Wuhan, China; ^2^ Department of Neurosurgery, Affiliated Hospital of Hubei University of Medicine, First People’s Hospital of Xiangyang, Xiangyang, China; ^3^ Department of Paediatrics, Xiangyang Central Hospital, Affiliated Hospital of Hubei University of Arts and Science, Central Hospital of Xiangyang, Xiangyang, China

**Keywords:** PFT, hydrocephalus, adult, evd, VP shunt

## Abstract

**Objective:**

This study aims to identify the predictors of postoperative hydrocephalus in patients with posterior fossa tumors (PFTs) and guide the management of perioperative hydrocephalus.

**Methods:**

We performed a single-institution, retrospective analysis of patients who underwent resection of PFTs in our department over a 10-year period (2011–2021). A total of 682 adult patients met the inclusion criteria and were divided into either a prophylactic external ventricular drainage (EVD) group or a nonprophylactic-EVD group. We analyzed data for the nonprophylactic-EVD group by univariate and multivariate analyses to identify predictors of postoperative acute hydrocephalus. We also analyzed all cases by univariate and multivariate analyses to determine the predictors of postoperative ventriculoperitoneal (VP) shunt placement.

**Results:**

Tumor infiltrating the midbrain aqueduct [*P* = 0.001; odds ratio (OR) = 9.8], postoperative hemorrhage (*P* < 0.001; OR = 66.7), and subtotal resection (*P* = 0.006; OR = 9.3) were independent risk factors for postoperative EVD. Tumor infiltrating the ventricular system (*P* < 0.001; OR = 58.5) and postoperative hemorrhage (*P* < 0.001; OR = 28.1) were independent risk factors for postoperative VP shunt placement.

**Conclusions:**

These findings may help promote more aggressive monitoring and earlier interventions for postoperative hydrocephalus in patients with PFTs.

## Introduction

Resection is the primary treatment measure for patients with posterior fossa tumors (PFTs), and hydrocephalus is a common perioperative complication (1, 2). Postoperative acute hydrocephalus often increases the length and cost of hospitalization and negatively affects the patient’s prognosis. Preoperative external ventricular drainage (EVD) and endoscopic third ventriculostomy (ETV) have been suggested to prevent postoperative acute hydrocephalus (3–5); however, the possibilities of additional brain-tissue damage and complications such as intracranial infection and hemorrhage also need to be considered (3, 6–9). Some studies found that the incidence of postoperative hydrocephalus in adult patients with PFTs was less than in juvenile PFT patients (10, 11) and hydrocephalus resolved in 96% of adult patients after tumor resection (2). Identifying adult patients at high risk of postoperative hydrocephalus and the placement of a prophylactic EVD is thus still controversial (3, 12, 13). Many studies have focused on this issue in pediatric patients (13–17), but few studies have considered adult patients (18). This study aimed to investigate the predictors of acute and persistent hydrocephalus development after resection in adult patients with PFTs.

## Materials and Methods

### Study Population and Data Collection

This retrospective descriptive cohort study investigated the incidence and predictors of postoperative acute and persistent hydrocephalus in consecutive patients who underwent surgery for PFT between January 2011 and February 2021 at the Department of Neurosurgery, Zhongnan Hospital of Wuhan University. A total of 762 patients aged >18 years who had previously been diagnosed with PFTs on the basis of outpatient computerized tomography (CT) or magnetic resonance imaging (MRI) were admitted to our department. The exclusion criteria were patients who underwent biopsy or did not receive tumor resection, patients who had a ventriculoperitoneal (VP) shunt or ETV performed before resection, and patients with a diagnosis of brain abscess. Clinical information was recorded for all enrolled patients, and the follow-up duration ranged from 90 days to 6 years.

Follow-up data were obtained from the last outpatient consultation or via telephone calls with patients if the follow-up was not appropriate for the specific patient. The data collected in this study were obtained from the hospital’s electronic database. The following information was recorded: patient characteristics, surgical procedure, placement of perioperative EVD, ETV, and VP shunt, and histological results. Tumor growth characteristics, tumor size, and the presence of preoperative hydrocephalus were determined via preoperative MRI scans. The extent of resection, postoperative hemorrhage, and presence of postoperative hydrocephalus was determined by postoperative CT and/or MRI scans.

The diagnostic criteria for preoperative hydrocephalus were imaging results indicating Evans’ index (maximum width between frontal horns divided by maximal width of inner table) >0.3 with signs of intracranial hypertension such as headache, nausea, and visual deterioration (19).

#### Prophylactic EVD Placement

The prophylactic EVD used in this study aimed to treat preoperative hydrocephalus, relieve intracranial hypertension, and prevent damage caused by acute hydrocephalus after resection. We considered both preoperative and intraoperative EVD as prophylactic EVD. The placement of an EVD device was decided by surgeons after a discussion of the case. The criteria for preoperative EVD placement were a radiographic diagnosis of hydrocephalus with severe clinical symptoms, such as frequent vomiting and a decreased level of consciousness at admission due to intracranial hypertension caused by hydrocephalus. If the patient’s decreased consciousness was caused by a brain stem tumor, cerebral herniation, or intracranial hypertension resulting from venous sinus compression, we usually chose to remove the lesion directly. However, in patients with mild clinical symptoms of intracranial hypertension, such as headache and nausea, we aimed to complete the preoperative preparation and perform surgery as soon as possible to relieve intracranial hypertension. We placed an intraoperative EVD as soon as the surgical procedure became difficult as a result of severe brain swelling.

#### Postoperative EVD Placement

The criteria for postoperative acute hydrocephalus were Evans’ index >0.3 and symptomatic intracranial hypertension leading to vomiting, nausea, or impaired consciousness (20). All patients with postoperative acute hydrocephalus received an EVD in our department. The time from EVD insertion to drainage removal was <14 days in all cases. The criteria for removal of the drain were patient remaining in stable condition while increasing the drainage height for several days and then closing the drain for at least 12 h and no evidence of hydrocephalus on CT images for 24 h before drain removal.

#### Postoperative VP Shunt Placement

The criterion for placement of a postresection VP shunt was failed EVD weaning or symptomatic persistent hydrocephalus necessitating permanent drainage.

#### Tumor Growth Characteristics

**Figure 1:**
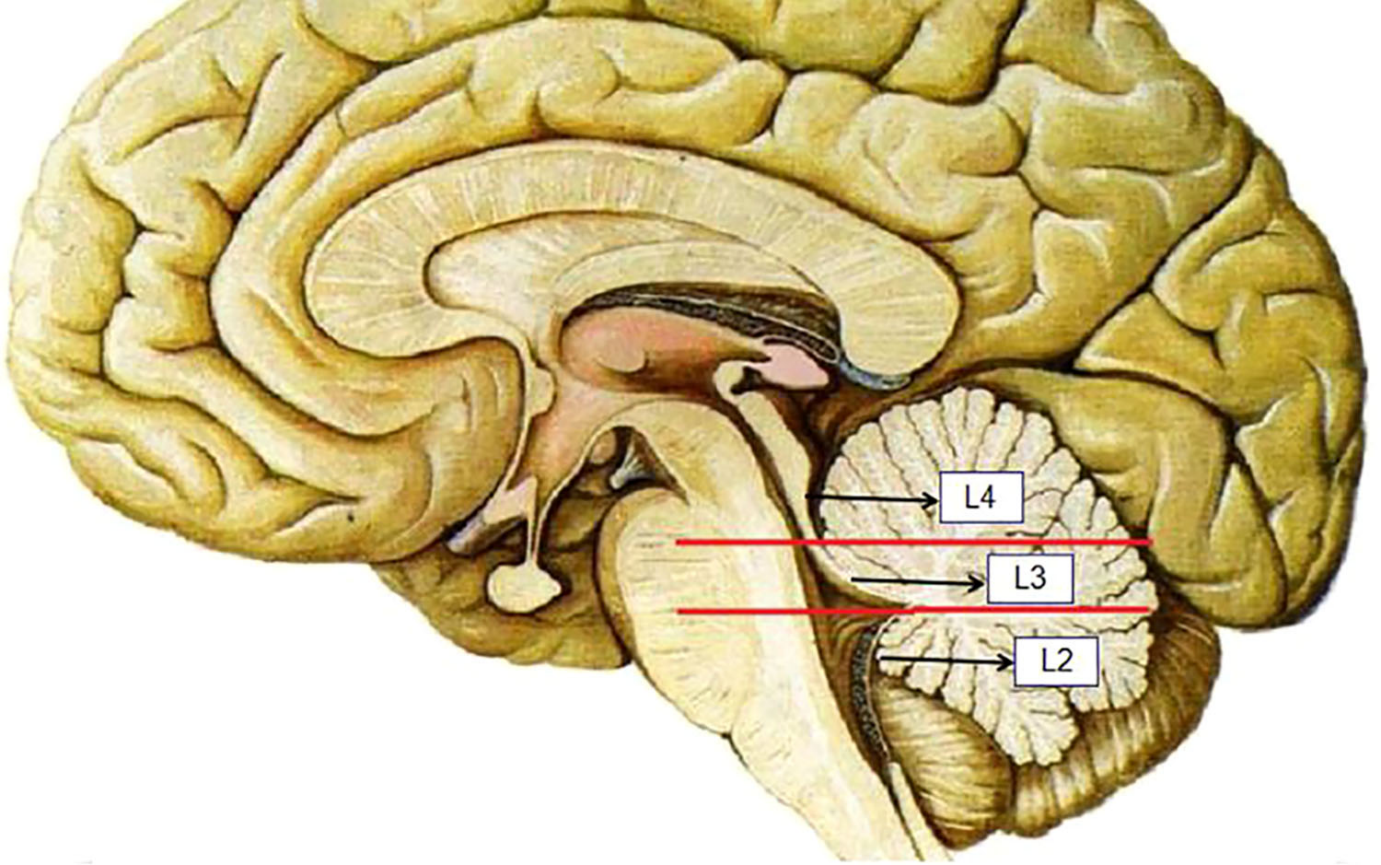
Section of L2, L3, and L4 according to the infiltration height of the tumor in the subtentorial ventricular system.

**Figure 2:**
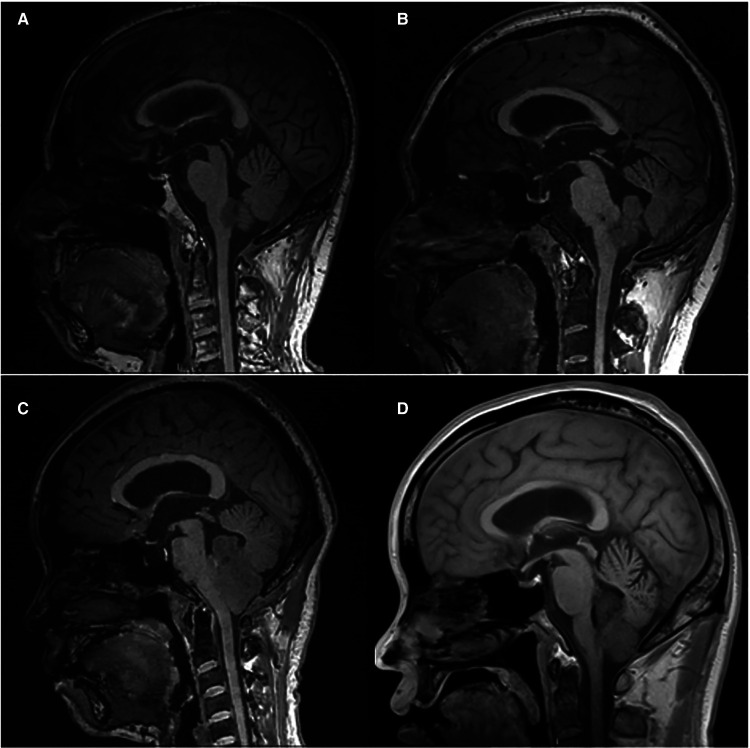
(**A**) and (**B**): sagittal (T1) views of type L2 tumor, (**C**) and (**D**): sagittal (T1) views of type L3 tumor.

**Figure 3:**
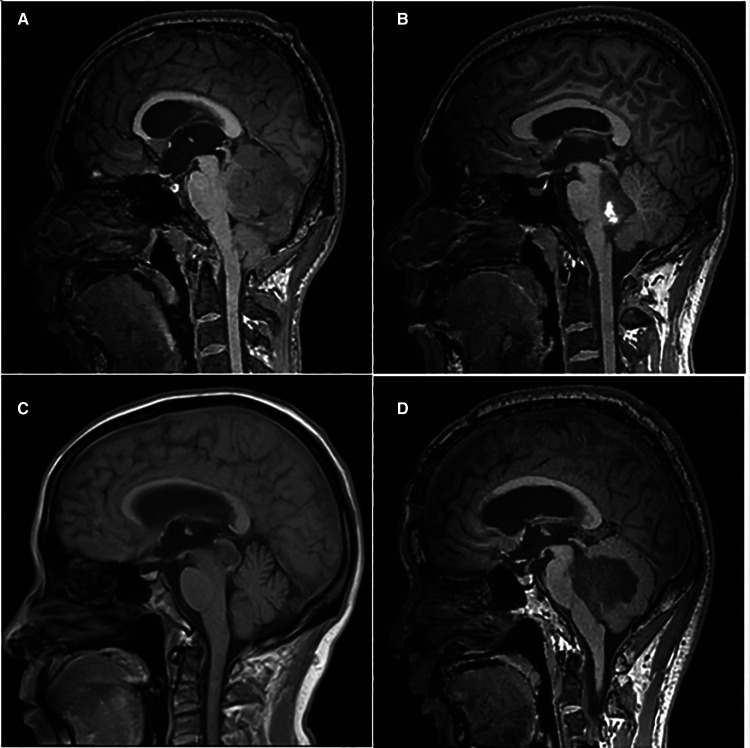
(**A**): sagittal (T1) view of the tumor infiltration from the periphery; (**B**): sagittal (T1) view of the tumor infiltration from below; (**C**): sagittal (T1) view of the tumor endogenous from the ventricular system; (**D**): sagittal (T1) view of the tumor infiltration of the upper half of the fourth ventricle.

The ventricular system comprises a set of adjoining cavities that produce and circulate cerebrospinal fluid (CSF) within the brain (21). Ventricular infiltration was defined as a tumor infiltrating the brain tissue around the CSF circulation channel or invading the ventricular channel. In terms of spatial morphology, the CSF pathway tends to increase from the lower part upward to the lower half of the fourth ventricle and then decrease from the upper part of the fourth ventricle upward to the midbrain aqueduct. In addition, differences exist during surgery, with most type L2 tumors removable by elevating the cerebellum, while most type L3 and type L4 tumors require cerebellum incision. Based on the above characteristics and preoperative MRI scans, we classified each patient into the corresponding group according to the infiltration height of the tumor in the subtentorial ventricular system (Figure 1) as follows: L1, extra-axial tumor not infiltrating the ventricular system; L2, tumor infiltrating the ventricular system up to the lower half of the fourth ventricle (Figures 2A, B); L3: tumor infiltrating the ventricular system up to the upper half of the fourth ventricle (Figures 2C, D, 3D); and L4: tumor infiltrating the ventricular system up to the midbrain aqueduct, with invasion from the periphery (Figure 3A), invasion from below (Figure 2B), or endogenous tumor of the midbrain aqueduct (Figure 3C).

#### Extent of Resection

Total resection was defined as no residual tumor tissue seen by surgeons after resection and no reactive enhancement within 72 h after surgery on MRI (22). Tumor size was calculated using the maximum cross-sectional area of the tumor on MRI scans.

#### Postoperative Hemorrhage

Postoperative hemorrhage was diagnosed from postoperative CT images. For patients with postoperative hemorrhage not flooding into the ventricle, we performed evacuation of the hematoma for patients with massive hemorrhage. For patients with postoperative intraventricular hemorrhage, we placed an EVD with or without evacuation of the intracranial hematoma.

#### Surgical Position

Patients with midline PFTs undergoing surgery at our center primarily adopted a semi-sitting position if a relevant patent foramen ovale was not detected, while patients with nonmidline PFTs primarily adopted a supine position.

### Study Design

Patients were classified into either a nonprophylactic-EVD group or a prophylactic-EVD group. The primary and secondary endpoints of the study were to determine the predictors of postoperative acute hydrocephalus and persistent hydrocephalus, respectively. The age and tumor size were analyzed as median values. Tumor growth characteristics were divided into four categories, and each patient was assigned to one respective group to rule out bias. We dichotomized other clinical and radiological parameters. EVD was placed in all patients with postoperative acute hydrocephalus, and all enrolled patients who developed postoperative persistent hydrocephalus received a VP shunt. We analyzed the nonprophylactic-EVD group to identify the predictors of postoperative acute hydrocephalus to eliminate bias caused by other perioperative CSF drainage. We also analyzed all cases to determine the predictors of postoperative persistent hydrocephalus. In view of the small number of patients who received VP shunts, we reduced the stratification and included types L2, L3, and L4 tumors in one group of tumors infiltrating the ventricular system to reduce the bias.

This study was approved by the Ethics Committee of Zhongnan Hospital of Wuhan University. The study did not involve patients’ personal or private information, and patient consent was therefore not required.

### Statistical Analysis

Data analysis was performed using IBM SPSS Statistics (IBM releases 22 and 24). Parameters were compared between groups using Student’s *t*-test. Binary parameters were analyzed by *χ*^2^ tests. Multivariate logistic regression analysis was performed to identify independent predictors of EVD placement. Odds ratios (ORs) and 95% confidence intervals were calculated to assess the impacts of the variables. A *P*-value <0.05 was considered statistically significant.

Data Availability Statement: Data for this study will be provided upon reasonable request.

## Results

### Patient Characteristics

**Figure 4:**
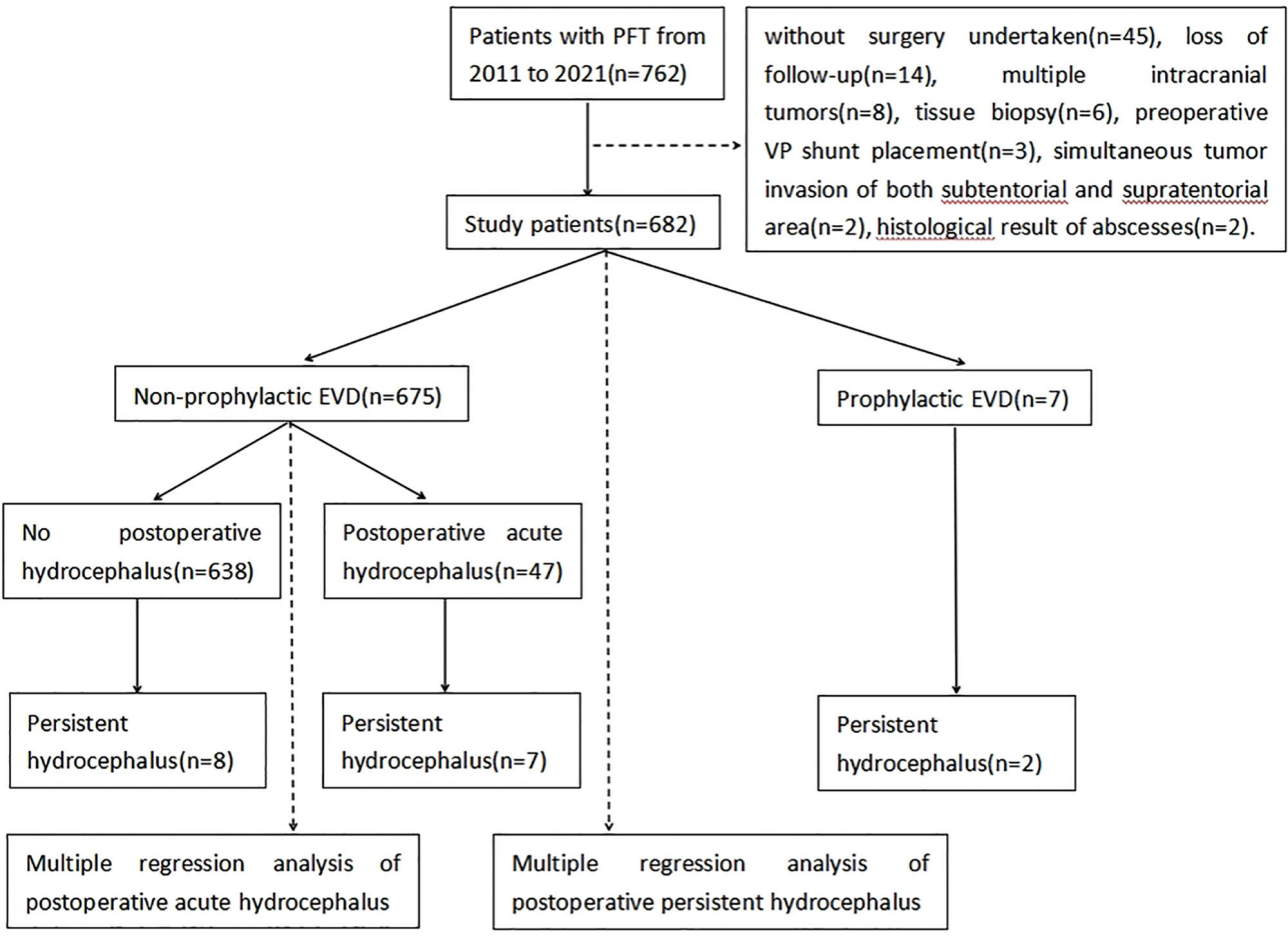
Flow chart for the study population.

A total of 682 of 762 patients were finally enrolled in this study. Eighty patients were excluded in accordance with the inclusion criteria, including 42 patients without surgery, 14 patients who were lost to postoperative follow-up, eight patients with multiple intracranial tumors, six patients with biopsies, three patients with direct gamma-knife treatment, three patients with a preoperative VP shunt, two patients with simultaneous tumor invasion of both the subtentorial and supratentorial areas, and two patients with histological brain abscesses (Figure 4).

The average age of the 682 patients was 48.6 years (range 19–83 years). There were 272 (31%) men and 410 (69%) women. The pathological findings included 570 (84%) cases of benign tumors and 112 (16%) cases of malignant tumors.

Seven of the enrolled patients (1%) received prophylactic EVD, including six patients with preoperative hydrocephalus who received preoperative EVD for severe intracranial hypertension and one patient without preoperative hydrocephalus who received EVD because of intraoperative hemorrhage and brain swelling. Two of the seven patients received a postoperative VP shunt due to EVD-weaning failure. Patient information is shown in Table 1.

**Table 1:** Details of the prophylactic EVD group.

Sex	Age (years)	Size (cm²)	Growth characteristics	Total resection	Postoperative hemorrhage	Preoperative hydrocephalus	Postoperative VP shunt	Histology
Female	25	16.3	L1	Yes	No	Yes	No	Schwannoma
Female	19	15.7	L4	Yes	No	Yes	No	Choroid plexus papilloma
Female	39	1.5	L3	No	Yes	No	Yes	Ganglioglioma
Female	27	23.2	L3	Yes	Yes	Yes	Yes	Medulloblastoma
Male	67	12	L3	No	No	Yes	No	Astrocytoma
Male	55	30.6	L2	Yes	No	Yes	No	Astrocytoma
Male	42	18.4	L3	No	No	Yes	No	Astrocytoma

*EVD, external ventricular drainage; VP, ventriculoperitoneal.*

A total of 136/675 (20%) patients presented with preoperative hydrocephalus. The symptoms of intracranial hypertension were resolved or alleviated in 113 of these 136 (83%) patients with preoperative hydrocephalus without prophylactic EVD placement. A total of 47/675 (7%) patients developed postoperative acute hydrocephalus, and 9 (19%) of these patients received a VP shunt due to EVD-weaning failure. In addition to these 47 patients, 8 of the 628 (13%) patients subsequently developed chronic hydrocephalus and received a VP shunt. Finally, 55 patients developed hydrocephalus and received an EVD or/and VP shunt placement postoperatively. Twenty-four of 55 (44%) patients with postoperative hydrocephalus had ventricular system infiltration. Demographic data and clinical characteristics of patients with PFTs are shown in Table 2.

**Table 2:** Demographic and clinical characteristics of patients with PFTs.

	Total	Malignant	Postoperative hydrocephalus					
			Cerebellar	Ventricle	Brainstem	Tentorial	Cerebellopontine	Ventricular system infiltration
Schwannoma	287	0	0	0	0	0	18/287	1/2
Meningoma	132	4	3/52	0	0	2/17	2/63	1/3
Hemangioblastoma	93	0	7/79	0	2/8	2/6	0	3/6
Glioma	63	40	4/56	3/3	3/4	0	0	9/10
Metastasis	29	29	0/27	1/2	0	0	0	1/2
Ependymoma	17	16	0	3/17	0	0	0	3/17
Medulloblastoma	9	9	0	3/9	0	0	0	3/9
Choroid plexus Papilloma	8	0	0	2/8	0	0	0	2/8
Arachnoid cyst	2	0	0	1/2	0	0	0	1/2
Cholesteatoma	17	0	0/2	0	0	0	0/15	0
Epidermoid cyst	9	0	0/1	0	0	0	0/8	0
Solitary fibrous tumor	12	12	0	0	0	0	0/12	0
Lymphoma	2	2	0/2	0	0	0	0	0
Teratoma	2	0	0/2	0	0	0	0	0
Total	682	112	14/221	13/41	5/12	4/23	20/385	24/59

*PFT, posterior fossa tumor*.

### Predictors of Postoperative Acute Hydrocephalus

The nonprophylactic-EVD group included 675 patients [average age 48.6 years, range 19–83 years; 269 (40%) men and 406 (60%) women]. A total of 565 (84%) patients had benign tumors and 110 (16%) patients had malignant tumors; 23/136 (17%) patients with preoperative hydrocephalus received postoperative EVD due to unresolved or aggravated symptomatic hydrocephalus. Newly emerging acute hydrocephalus developed in 24/539 (4%) patients. The mean EVD-implantation time was 2.4 days (range 1–10 days). The highest morbidity rates in this group occurred in patients with schwannomas (42%) and meningiomas (20%). The rate of preoperative hydrocephalus was highest in patients with medulloblastomas (88%) and ependymomas (71%), while the rate of postoperative acute hydrocephalus was the highest in patients with choroid plexus papillomas (29%) and gliomas (15%). The rates of new acute hydrocephalus were the highest in patients with choroid plexus papillomas (29%) and hemangiomas (8%). The tumor type and incidence of perioperative hydrocephalus in the nonprophylactic-EVD group are shown in Table 3.

**Table 3:** Details of the nonprophylactic-EVD group.

	Value	Preoperative hydrocephalus	Postoperative hydrocephalus	New Postoperative hydrocephalus
Benign	565(84%)	90(16%)	35(6%)	23(4%)
Malignant	110(16%)	46(42%)	12(11%)	1(1%)
Schwannoma	286(42%)	42(15%)	17(6%)	12(4%)
Meningoma	132(20.0%)	25(18.9%)	7(5.3%)	2(2%)
Hemangioblastoma	93(14%)	14(15%)	9(9%)	7(7%)
Glioma	59(9%)	18(31%)	9(15%)	1(2%)
Metastasis	29(4%)	9(31%)	1(3%)	0
Ependymoma	17(3%)	12(71%)	0	0
Cholesteatoma	17(3%)	1(6%)	0	0
Solitary fibrous tumor	12(2%)	1(8%)	0	0
Epidermoid cyst	9(1%)	1(11%)	0	0
Medulloblastoma	8(1%)	7(88%)	2(13%)	0
Choroid plexus papilloma	7(1%)	3(43%)	2(29%)	2(29%)
Arachnoid cyst	2(1%)	1(50%)	0	0
Lymphoma	2(1%)	1(50%)	0	0
Teratoma	2(1%)	1(50%)	0	0
Total	675	136(20.1%)	47(7.0%)	24(3.6%)

*EVD, external ventricular drainage.*

The incidence of postoperative acute hydrocephalus was 5% (32/594) in patients with tumors without ventricular system infiltration (L1), compared with 19% (15/81) in tumors with ventricular system infiltration (L2, L3, L4). Interestingly, none of the six cases of type L2 developed postoperative acute hydrocephalus. The incidences of postoperative hemorrhage were 56% (18/32) in cases of type L1 with postoperative acute hydrocephalus and 33% (5/15) in tumors with ventricular system infiltration (L2, L3, L4).

Univariate analysis showed that postoperative EVD due to acute hydrocephalus was significantly correlated with tumor growth characteristics (*P* < 0.001), resection degree, postoperative hemorrhage (*P* < 0.001), and preoperative hydrocephalus (*P* < 0.001) (Table 4). Multivariate analysis identified tumor type L4 (OR = 9.8), postoperative hemorrhage (OR = 66.7), and subtotal tumor resection (OR = 9.3) as independent risk factors for postoperative acute hydrocephalus (Table 5).

**Table 4:** Summary of the factors with postoperative EVD.

	Postoperative EVD		*P*- value
	Yes	No	
Sex			0.06(Pearson’s chi-square)
Male	25(53%)	244(39%)	
Female	22(47%)	384(61%)	
Age (years)	52.4 ± 7.1	49.1 ± 11.9	0.31(Student’s *t*-test)
Tumor size (cm^2^)	9.7 ± 3.3	8.5 ± 2.1	0.19(Student’s *t*-test)
Growth characteristics			<0.001(Pearson’s chi-square)
L1	32(68%)	562(90%)	
L2	2(4%)	50(8%)	
L3	0	6(1%)	
L4	13(28%)	10(2%)	
Postoperative hemorrhage			<0.001(Pearson’s chi-square)
Yes	19(40%)	7(1%)	
No	28(60%)	621(99%)	
Preoperative hydrocephalus			<0.001(Pearson’s chi-square)
Yes	23(49%)	113(18%)	
No	24(51%)	515(82%)	
Total resection			<0.001(Pearson’s chi-square)
Yes	39(83%)	614(98%)	
No	8(17%)	14(2%)	
Histology			0.098(Pearson’s chi-square)
Benign	35(75%)	530(84%)	
Malignant	12(25%)	98(16%)	
Surgical position			0.005(Pearson’s chi-square)
Semi-sitting position	28(60%)	244(39%)	
Supine position	19(40%)	384(61%)	

*EVD, external ventricular drainage.*

**Table 5:** Univariate and multivariate analyses of the association between postoperative EVD and factors.^a^

	Univariate analysis		Multivariate analysis	
	*P* value	OR(95%CI)	*P* value	OR(95%CI)
Growth characteristics	0.001	-	0.004	-
L2	0.36	0.51(0.12–2.18)	0.81	-
L3	0.501	0.5	1	-
L4	0.001	23.63(9.67–57.76)	0.01	9.8(2.1–45.6)
Postoperative hemorrhage	0.001	60.20(23.38–154.99)	<0.001	66.7(22.2–185.2)
Preoperative hydrocephalus	0.001	0.23(0.13–0.42)	0.17	-
Subtotal resection	0.001	9.0(3.56–22.73)	0.006	9.3(1.9–45.5)
Semi-sitting position	0.005	2.3 (1.27–4.24)	0.79	-

^a^ *Binary logistic regression with stepwise exclusion was used for multivariate analysis; Nagelkerke R^2^ = 0.428*

*EVD, external ventricular drainage; OR, odds ratio.*

### Predictors of Postoperative VP Shunt

Overall, 17/682 (3%) patients received postoperative VP shunt placement, including two (12%) patients who initially received postoperative ETV, followed by VP shunt placement for unresolved obstructive hydrocephalus. The mean implantation time was 62.4 days (range 14–191 days). In univariate analysis, postoperative VP shunt placement due to persistent hydrocephalus was significantly correlated with tumor growth characteristics (*P* < 0.001), postoperative hemorrhage (*P* = 0.001), preoperative hydrocephalus (*P* = 0.001), subtotal resection (*P* = 0.001), tumor pathology (*P* = 0.005), and surgical position (*P* = 0.04) (Table 6).

**Table 6:** Summary of the factors with postoperative VP shunt placement.

	Postoperative VP shunt		*P* value
	Yes	No	
Sex			0.54(Pearson’s chi-square)
Male	8(47%)	264(40%)	
Female	9(53%)	401(60%)	
Age (years)	47.2 ± 13.92(23–83)	49.5 ± 10.44(19–82)	0.5(Student’s *t*-test)
Tumor size (cm^2^)	7.5	8.7	0.92(Student’s *t*-test)
Tumor infiltrating the ventricular system			<0.001(Pearson’s chi-square)
Yes	15(88%)	72(11%)	
No	2(12%)	593(89%)	
Postoperative hemorrhage			<0.001(Pearson’s chi-square)
Yes	8(47%)	20(3%)	
No	9(53%)	645(97%)	
Preoperative hydrocephalus			0.001(Pearson’s chi-square)
Yes	10(59%)	132(20%)	
No	7(41%)	533(80%)	
Total resection			<0.001(Pearson’s chi-square)
Yes	10(59%)	647(97%)	
No	7(41%)	18(3%)	
Prophylactic EVD			<0.001(Fisher’s exact test)
Yes	2(12%)	5(1%)	
No	15(88%)	660(99%)	
Histology			0.005(Pearson’s chi-square)
Benign	10(59%)	560(84%)	
Malignant	7(41%)	105(16%)	
Surgical position			0.041(Pearson’s chi-square)
Semi-sitting position	11(65%)	266(40%)	
Supine position	6(35%)	399(60%	

*VP, ventriculoperitonea; EVD, external ventricular drainage*.

In multivariate analysis, both tumor infiltrating the ventricular system (OR = 58.5) and postoperative hemorrhage (OR = 28.1) were independent risk factors for postoperative VP shunt placement (Table 7).

**Table 7:** Univariate and multivariate analyses of the association between postoperative VP shunt placement and factors.^a^

	Univariate analysis		Multivariate analysis	
	*P* alue	OR(95%CI)	*P* value	OR(95%CI)
Tumor infiltrating the ventricular system	<0.001	-	<0.001	58.5(7.9–431.6)
Postoperative hemorrhage	0.001	28.67(10–82)	<0.001	28.1(4.6–171.8)
Preoperative hydrocephalus	0.001	5.8(2.2–15.4)	0.5	-
Total resection	0.001	0.04(0.01–0.12)	0.07	-
Malignant	0.005	3.7(1.4–10)	0.72	-
Prophylactic EVD	0.011	0.06(0.01–0.3)	0.75	-
Semi-sitting position	0.04	2.8(1–7.5)	0.24	-

^a^ *Binary logistic regression with stepwise exclusion was used for multivariate analysis; Nagelkerke R^2^ = 0.555*.

*VP, ventriculoperitonea; OR, odds ratio; EVD, external ventricular drainage*.

## Discussion

Postoperative symptomatic hydrocephalus is a severe complication after resection of PFT, but there is still no consensus on the placement of a prophylactic EVD. The current study evaluated the incidence of perioperative hydrocephalus in adult patients with PFTs, with the aim of identifying the predictors of postoperative hydrocephalus requiring CSF drainage, and determined the value of prophylactic EVD. The incidence of preoperative hydrocephalus in adult patients with PFTs in our study was 20.8% and the incidence of persistent postoperative hydrocephalus was 3.6%, compared with rates of 21.4% and 2.1%, respectively, reported by Marx et al. (2) The ratio is much higher in pediatric patients (1), largely due to the different distribution of PFTs by histology in pediatric and adult patients. In our study, schwannomas and meningiomas accounted for the highest proportions of PFTs. Numerous studies have shown that these tumors rank in the top three PFTs in terms of morbidity (2, 18, 23). The symptoms of intracranial hypertension can be resolved or alleviated in most patients with preoperative hydrocephalus, and ETV or EVD prior to PFT surgery is not routinely recommended in adult patients.

In our study, the incidences of preoperative hydrocephalus were the highest in patients with medulloblastomas and ependymomas. These tumors had similar incidences and ranked second after metastasis among all PFTs in the study by Marx et al. (2). This is related to the corresponding tumor growth characteristics, with an increased risk of obstructing the CSF circulation pathway as the tumor volume increases. Preoperative hydrocephalus was not a significant predictor of postoperative hydrocephalus requiring drainage in our study. This was in contrast to the study by Won et al., who found that pilocytic astrocytoma and preoperative hydrocephalus were independent risk factors for the development of postoperative hydrocephalus in adult patients (11).

The incidence of postoperative hydrocephalus was the highest in patients with choroid plexus papillomas (29%) in our research. Two of the seven patients with choroid plexus papilloma who did not receive prophylactic EVD developed acute obstructive hydrocephalus after resection due to postoperative hemorrhage and cerebral edema, and one of these two also received a VP shunt due to EVD-weaning failure. Previous research suggested that plexus papilloma tumors showed the most significant association with VP shunt dependence (24), given that these tumors often invade the ventricle compartments, and ventricle entry has been reported to increase the risk of postoperative hydrocephalus. However, the incidence of plexus papilloma tumors was not high in another study (2). However, the current and previous studies all included small numbers of these patients, and further studies with larger data volumes are needed to clarify this.

In our study, postoperative acute hydrocephalus was relatively common in patients with choroid plexus papillomas, gliomas, and medulloblastomas, all of which are located near the midline. The incidence of postoperative acute hydrocephalus was 5% in tumors without ventricular system infiltration compared with 19% for tumors infiltrating the ventricular system. The incidence of postoperative acute hydrocephalus was thus lower in tumors without ventricular system infiltration and was closely related to postoperative hemorrhage, which is known to increase the risk of hydrocephalus. Some studies have also suggested that the incidence of postoperative hydrocephalus is higher in tumors located near the midline (25). Chen et al. analyzed fourth ventricular tumors and considered the upward growth of tumors as an independent risk factor for postoperative CSF drainage (26). Tumor growth was a significant factor in the current univariate analysis, while type L4 tumors were an independent risk factor for postoperative acute hydrocephalus in multivariate analysis, using type L1 tumors as a reference. Type L2 tumors are anatomically distant from the midbrain aqueduct, and releasing CSF, elevating the cerebellum, or even sacrificing portions of cerebellar tissue during surgery can make it easier to obtain adequate surgical space. Although type L3 tumors are located adjacent to the midbrain aqueduct, the space of the midbrain aqueduct above the lesion is often dilated, secondary to intracranial hypertension. Compared with type L2 and L3 tumors, resection of type L4 tumors is more difficult and can cause more severe brain-tissue damage. In addition, the midbrain aqueduct is a relatively narrow part of the ventricular system anatomically and is thus more susceptible to stenosis or obstruction from postoperative brain swelling, residual hematoma formation, the mass effect, and migration of hemostatic materials (26).

Won et al. found that placing patients in a semi-sitting position could reduce the incidence of postoperative hydrocephalus (18). This position has the advantage of improving the outflow of blood and protein-rich CSF during surgery. However, the semi-sitting position was not an independent predictor of postoperative hydrocephalus in the current study. This difference may be due to their routine selection of the semi-sitting position, while we select this position for most midline tumors and partial nonmidline tumors.

Total resection was an independent protective factor for postoperative acute hydrocephalus in our study. Residual tumors may have a mass effect, which is particularly evident for tumors that infiltrate the ventricles. Clinically, tumors with unclear margins and a rich blood supply are more difficult to resect totally. Postoperative local cerebral edema around these tumors can be severe, and the risk of postoperative cerebral hemorrhage is higher, leading to a higher incidence of postoperative acute obstructive hydrocephalus. However, total resection is not appropriate for all PFTs. Won et al. suggested that total resection of intraparenchymal tumors was a protective factor for CSF drainage (18). Some studies have suggested that postoperative acute hydrocephalus is related to brain swelling caused by the delayed opening of the venous sinus after the recovery of blood supply to the compressed brain tissues (27, 28). For tumors without venous sinus infiltration or compression, total resection can provide more space to alleviate the effects caused by brain swelling. However, for tumors infiltrating the venous sinus, we often select subtotal resection because previous studies suggested that treating subtentorial meningiomas with total venous sinus resection was a risk factor for postoperative acute hydrocephalus (29, 30).

Postoperative hemorrhage is a serious complication, with a reported incidence in PFTs of 3%, most of which are substantial tumors (31). The incidence of postoperative hemorrhage in the current study was 4% and usually occurred within 24 h after surgery. Supratentorial hemorrhage has a mass effect and postoperative hemorrhage near the midline can block the CSF pathway, thus causing acute obstructive hydrocephalus. However, the incidence of postoperative hemorrhage can be effectively reduced by appropriate management of intracranial pressure, perioperative blood pressure, and abnormal coagulation. The incidence of postoperative persistent hydrocephalus in the present study was 2%, compared with 6% (11, 32) reported by Marx et al. and 10% by Won et al. (18).

Tumor infiltration of the ventricular system was an independent risk factor for VP shunt placement after resection, consistent with the conclusion that postoperative hydrocephalus is more likely to occur when tumors are located near the midline (13). This may be due to differences in tumor location and histological characteristics; compared with type L1 tumors, tumors infiltrating the ventricular system are usually associated with increased risks of intraventricular hemorrhage and postoperative hydrocephalus (33). Furthermore, recurrence of these tumors can be more prone to cause obstruction of the CSF pathway.

The current study did not consider if prophylactic EVD could reduce the occurrence of postoperative persistent hydrocephalus. Radiographic upward herniation is common in patients with obstructive hydrocephalus associated with a PFT. It is advisable to be cautious when placing a preoperative EVD in patients with a PFT causing radiographic upward herniation, and this intervention should be reserved for patients with severe symptomatic obstructive hydrocephalus, in whom tumor resection is not immediately feasible. It is also reasonable to maintain a relatively high level of drainage about the tragus to avoid sudden or excessive CSF outflow (34). This is one reason why prophylactic EVD is not generally placed in our center. Lumbar drainage placement is another controversial method of prophylactic CSF drainage. The potential risk of down-herniation cannot be ignored, and removal of the CSF from below the tentorium was proposed to increase the pressure gradient between the supratentorial and lumbar cistern compartments, promoting transtentorial herniation. Pneumocephalus enhanced this pressure gradient, thus causing clinical herniation syndrome (35). Excessive drainage due to EVD can cause localized hydrocephalus and increase the risk of shunt dependence (36). Some studies support the use of postoperative ETV, which has been reported to be effective in cases of EVD-weaning failure as an alternative to VP shunt placement (37). The success rate of ETV for the avoidance of shunt placement was 72% in adult patients with obstructive hydrocephalus due to aqueductal stenosis (38), which was higher than in pediatric patients (39); however, there is currently no consensus on this issue (40, 41). According to Srinivasan et al., the incidence of postoperative complications with ETV was 9%, and the rate of ETV failure was higher in postresection cases with posterior fossa ependymomas (42).

The propositions for early and direct VP shunt placement differ. We recommend initial EVD insertion when postoperative acute hydrocephalus occurs, but seven of 47 (15%) patients in the current study with postoperative acute hydrocephalus subsequently received a VP shunt due to EVD-weaning failure. The proposed mechanism of acute hydrocephalus involves increased resistance of CSF flow caused by local cerebral edema or blood clots in the ventricular system. In contrast, chronic hydrocephalus may be caused by persistent obstruction of the arachnoid granulations by blood-breakdown products and their inflammatory reactions (43). In patients with postoperative acute hydrocephalus, the incidence of persistent hydrocephalus can be reduced by using EVD to get through the cerebral edema period and drain intraventricular blood clots, thus avoiding the complications of shunt implantation. According to our institutional standards, no subdural, epidural, or intratumoral drainage tubes are placed after PFT resection, and repeated irrigation with saline after resection is therefore necessary to find the source of a hemorrhage and clear the residual blood before suturing.

The association between the degree of resection and the development of postoperative persistent hydrocephalus is controversial. Total resection was reported to reduce the occurrence of long-term postoperative hydrocephalus (44), possibly as a result of relieving the obstructive effect or slow growth kinetics. Re-resection can be selected in cases of symptomatic obstructive hydrocephalus caused by tumor recurrence.

## Limitations

This study had some limitations. First, total resection was a protective factor for postoperative persistent hydrocephalus in this study; however, the postoperative follow-up time varied from 3 months to 6 years, and it is therefore unclear if the patients with a short follow-up time might go on to develop hydrocephalus in the future. This might lead to potential bias in the results. Second, the study included few cases of ETV, and our ability to evaluate ETV was thus limited. Third, the prophylactic-EVD group only included seven patients. To reduce bias, we did not analyze this group separately, but we did include prophylactic EVD as a covariable in the analysis of all samples. Fourth, bias of surgical position selection based on the tumor location may have affected the analysis. Finally, differences in surgical procedures and perioperative management exist in different medical centers, which may influence the results.

## Conclusion

Postoperative acute hydrocephalus is associated with tumor infiltration of the midbrain aqueduct, which can be determined by preoperative imaging studies, and prophylactic EVD is recommended for patients with this tumor characteristic. Postoperative persistent hydrocephalus is associated with tumor infiltration of the upper part of the infratentorial ventricular system, and prophylactic EVD is useless in these cases, but postoperative EVD insertion can significantly decrease the incidence of shunt dependence in patients with postoperative acute hydrocephalus. Total resection and measures to avoid postoperative hemorrhage can reduce the incidences of postoperative acute and persistent hydrocephalus. The results of this study and the identified predictors may provide insights and help manage patients with PFTs.

## Data Availability

The raw data supporting the conclusions of this article will be made available by the authors without undue reservation.
